# Force fields of charged particles in micro-nanofluidic preconcentration systems

**DOI:** 10.1063/1.5008365

**Published:** 2017-12-21

**Authors:** Lingyan Gong, Wei Ouyang, Zirui Li, Jongyoon Han

**Affiliations:** 1 Institute of Laser and Optoelectronic Intelligent Manufacturing, College of Mechanical and Electrical Engineering, Wenzhou University, Wenzhou 325035, P.R. China; 2 Department of Electrical Engineering and Computer Science, Massachusetts Institute of Technology, Cambridge, Massachusetts 02139, USA; 3 Department of Biological Engineering, Massachusetts Institute of Technology, Cambridge, Massachusetts 02139, USA

## Abstract

Electrokinetic concentration devices based on the ion concentration polarization (ICP) phenomenon have drawn much attention due to their simple setup, high enrichment factor, and easy integration with many subsequent processes, such as separation, reaction, and extraction etc. Despite significant progress in the experimental research, fundamental understanding and detailed modeling of the preconcentration systems is still lacking. The mechanism of the electrokinetic trapping of charged particles is currently limited to the force balance analysis between the electric force and fluid drag force in an over-simplified one-dimensional (1D) model, which misses many signatures of the actual system. This letter studies the particle trapping phenomena that are not explainable in the 1D model through the calculation of the two-dimensional (2D) force fields. The trapping of charged particles is shown to significantly distort the electric field and fluid flow pattern, which in turn leads to the different trapping behaviors of particles of different sizes. The mechanisms behind the protrusions and instability of the focused band, which are important factors determining overall preconcentration efficiency, are revealed through analyzing the rotating fluxes of particles in the vicinity of the ion-selective membrane. The differences in the enrichment factors of differently sized particles are understood through the interplay between the electric force and convective fluid flow. These results provide insights into the electrokinetic concentration effect, which could facilitate the design and optimization of ICP-based preconcentration systems.

Analysis of low-abundance species in microliter or nanoliter-scale samples is one of the important tasks in chemical, biomedical and environmental applications.^1,2^ An essential step in such analysis involves the preconcentration of analytes, which facilitates subsequent procedures such as separation, collection, detection, reactions *etc*. In the past two decades, many microfluidics-based preconcentration techniques have been developed,^3^ such as field amplified sample stacking (FASS),^4,5^ isoelectric focusing (IEF),^6^ electric field gradient focusing (EFGF),^7^ dielectrophoretic trapping (DEPT),^8,9^ and electrokinetic trapping (EKT)^10,11^*.* Among these techniques, preconcentration utilizing the ion concentration polarization (ICP) phenomenon was one of the most efficient.^12^ As first proposed by Wang et al., a typical H-shaped micro-nanofluidic preconcentration device consists of two microchannels connected by a nanochannel array (see Fig. 1(a)).^5^ By setting up an electric field **E**_**N**_ across the negatively charged nanochannels and thus initiating selective ion transport, an ion depletion (ID) zone is formed in the upper channel near the micro-nanochannel interface.^13^ In the meantime, the application of a tangential electric field **E**_**T**_ upon the quasi-equilibrium space charges in electric double layer (EDL) near the charged wall generates electroosmotic flow (of the first kind, EOF1),^14^ which brings charged particles into the microchannel.^15^ When the charged particles arrive at the boundary of depletion zone, a strong electric force imposes a barrier on the migration of the particles. Consequently, particles are focused at the front of the barrier. Enrichment factors of 10^6^∼10^8^ have been achieved for trapping small proteins or DNA molecules in such devices.^10^

**FIG. 1.**
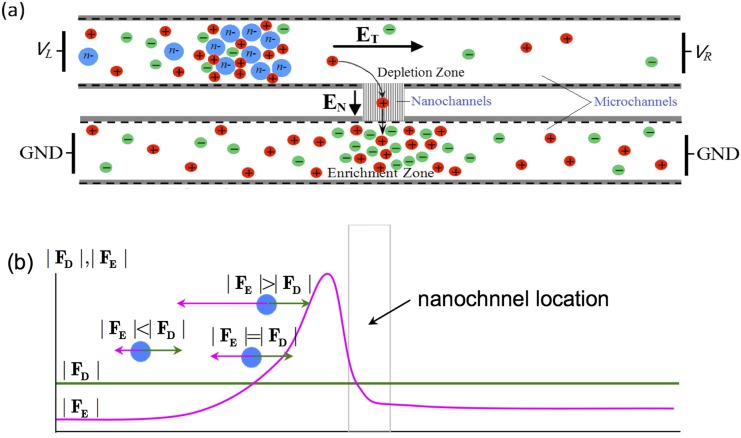
(a) Schematic of H-shape micro-nanofluidics preconcentration system; (b) Forces on the particles in 1D description.

The principle of the typical ICP-based micro-nanofluidic preconcentration is shown in Fig. 1(b). Assuming negatively charged microchannel walls, in the presence of a rightward **E**_**T**_, EOF runs from the left to the right, which applies a rightward drag force **F**_**D**_ on the particles. Meanwhile, for negatively charged particles, the electric force **F**_**E**_ on the particles points to the left. In the 1D description, in order for the particle to enter the microchannel, the magnitude of **F**_**D**_ must be greater than that of **F**_**E**_ at the inlet, while in the depletion zone, the former has to be smaller than the latter. At the locations where **F**_**D**_ and **F**_**E**_ are balanced, the particles are focused. Because the fluid flow velocity must be constant in a 1D channel, changing of the force balance has to be realized through the variation of the electric field.^16^

Although the 1D model above provides qualitative description of the micro-nanofluidic preconcentrator, the actual concentration system behaves far from 1D. The most important phenomenon absent in 1D model is the fast rotating vortices, induced by the electroosmotic flow of the second kind (EOF2) in the depletion zone near the membrane surface,^14,16–18^ which sends the fluid back into the upstream and expands the ID zone at the center of the channel. Experimentally, it has been frequently observed that the particles with low electrical mobility (e.g. proteins) shows focusing bands with oscillating protrusions near the channel walls into the depletion zone (Fig. 2(a), see Kim *et. al* for the experimental setup),^19^ while those with high electrical mobility (e.g. DNA) shows plug-like profiles (Fig. 2(b)). Meanwhile, it was found that the focusing bands of particles with low electrical mobility are very unstable, and the enrichment factor is far below that predicted from the 1D model. Reasons behind these phenomena require full, accurate analyses of two-dimensional (2D) distributions of forces, which however is very difficult to conduct through experimental observations.

**FIG. 2.**
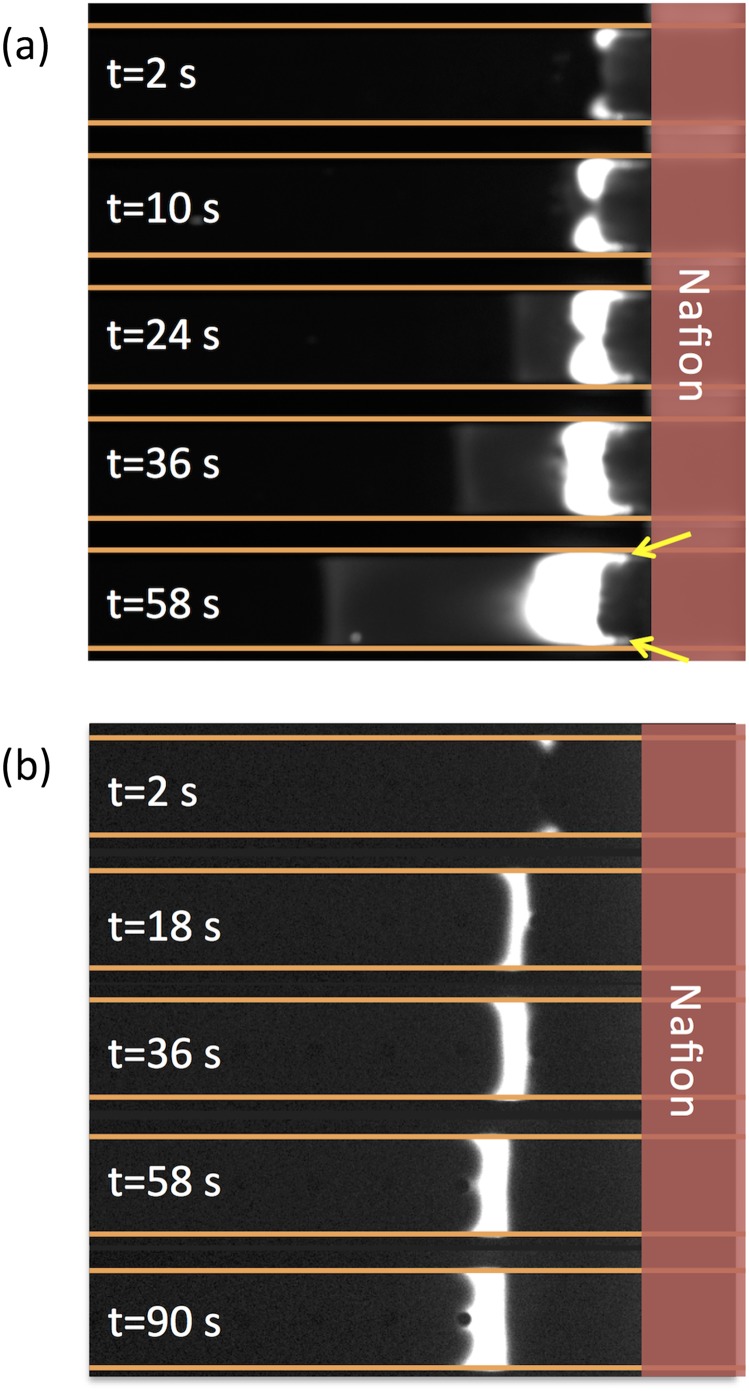
Evolution of the focusing band of (a) bovine serum albumin (BSA) protein and (b) a 22mer ssDNA in a nafion-based H-shaped preconcentrator (with only on CEM on one side). The BSA protein and ssDNA were labeled by Alexa Fluor 488 (with different degrees of labeling, the fluorescence intensity are not directly comparable between BSA and ssDNA). The yellow arrows in Fig. 2(a) show the protrusions of the focusing band into the depletion zone near the channel walls. The microchannel is 21μm deep, 200μm wide and 15mm long. The inlet and outlet of the upper channel are biased to 30V and 10V, respectively, while the lower channel is grounded. The background electrolyte is 0.1x phosphate buffered saline (PBS). The detailed experimental setup is described in Ref. 19.

The aim of this letter is to study the mechanisms of electrokinetic trapping that are not available in 1D description through 2D numerical simulations. By illustrating the interplay between the electric forces and fluid drag forces in the particle fluxes in 2D space, we will show that the enrichment factor is determined by the portion of particles that are sent to the rotating vortices at the vicinity of the membrane. Larger particles are less concentrated, because a larger portion of them *sneaks* through the membrane region without entering the vortex. The vortex flow strongly affects the motion of large particles, giving rise to the unstable bands with *protrusions* into the depletion zone near the channel walls. In addition, we also revealed that both the electric field and the fluid flow field are significantly distorted by the focused particles.

Fig. 3 shows the simulation model for the 2D micro-nanofluidic preconcentrator. The key component is a microchannel of length *L*=120 μm and width *H*=4 μm. In the middle of both the top and bottom walls, cation selective membranes (CEM) of length *L*_*m*_=2 μm are embedded. At both ends of the microchannel, compartments of width *L*_*1*_=60 μm and height *H*_*1*_=60 μm are connected. The microchannel walls are negatively charged with density σ_-_=-1mC/m^2^. The electrolyte solution in the reservoir outside the left boundary is a mixture of potassium chloride (KCl) buffer and negatively charged particles of valence *n-* (P^*n-*^). The diffusion coefficient of K^+^ and Cl^-^ are *D*_1_=1.957×10^-5^ cm^2^/s and *D*_2_=2.032×10^-5^ cm^2^/s, respectively. The electric potentials at the left and the right boundaries are set to *V*_*L*_=550 mV and *V*_*R*_=250 mV, respectively. The electric potentials at the CEM surfaces are set to zero. The charge density of CEM is assumed to be -2 mM, which is imposed by the boundary condition for K^+^ concentration that *C*_1_=2 mM on the CEM surfaces.^20,27^ The particles considered are BSA (*n*=14 at neutral pH, which were calculated using the protein sequence in NCBI Protein Library^21^ and the Protein Calculator (v3.4),^22^ diffusion coefficient 1.011×10^-7^ cm^2^/s)^23^ and a 22mer ssDNA (*n*=22 at neutral pH, diffusion coefficient 4.516×10^-7^ cm^2^/s).^24^ The channel in this model corresponds to the upper channel in Fig. 1(a), with the nanochannels replaced by CEM for simplicity. The symmetric setup (two CEMs on two sides) of the model resembles the H-shaped device in 3D experiments where the Nafion membrane spans across the bottom of the microchannel (Fig. 2), hence both the upper and lower microchannel walls of the H-shaped device are connected to the CEM. The lower channel in Fig. 1(a) where ion enrichment occurs has no effects on the simulation, so it is not included in the model. Besides, to save computing resources, only the lower half of the channel shown in Fig. 3 is computed with the symmetry boundary condition on the centerline of the channel.

**FIG. 3.**
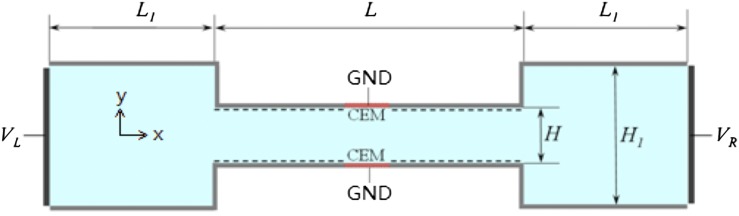
Simulation model for the micro-nanofluidic preconcentration system.

The governing equations for the incompressible fluid flow, ion and particle transport, and electric potential are described by the Navier-Stokes, Nernst-Planck, and Poisson equations, respectively:^25–27^

$$\rho \left(\partial U/\partial t+\left(U\cdot \nabla \right)U\right)=-\nabla P+\eta \nabla \cdot \nabla U-\rho_{e}\nabla \Phi ,$$ (1)

∇⋅U=0. (2)

$$\frac{\partial C_{i}}{\partial t}=-\nabla \cdot J_{i},$$ (3)

$$J_{i}=-\left(D_{i}\nabla C_{i}+Z_{i}\left(D_{i}F/RT\right)C_{i}\nabla \Phi \right)+UC_{i}.$$ (4)

$$-\nabla \cdot \left(\varepsilon \nabla \Phi \right)=\rho_{e}.$$ (5)

In Eq. 1 and Eq. 2, **U** ≡ (*u*, *v*) is the velocity; *P* is the pressure; Φ is the electric potential ($E\equiv \left(E_{x},E_{y}\right)=-\nabla \Phi$ is the electric field); *ρ*_*e*_ is the space charge density; constants *ρ* and *η* are the mass density and dynamic viscosity of the solution, respectively. In Eq. 3 and Eq. 4, *D*_*i*_, *Z*_*i*_, *C*_*i*_ and **J**_**i**_ ≡ (*J*_*i*,*x*_, *J*_*i*,*y*_) are the diffusion coefficient, valence, concentration and flux density of species *i*, respectively. For convenience, we use *i*=1 for K^+^, *i*=2 for Cl^-^, and *i*=3 for P^*n-*^. *T* is the absolute temperature. Constants *F* and *R* are the Faraday’s number and gas constant, respectively. In Eq. 5, *ε* is the permittivity of the solution. The space charge density is given by $\rho_{e}=e\sum_{i=1}^{3}Z_{i}C_{i}$, with *e* being the elementary charge. The boundary conditions are as follows:

At the membrane surfaces, it is assumed that: (1) fluxes of anions and particles across the membrane are zero; (2) the concentration of cations at the membrane surface is 2 mM; (3) the electric potential at the membrane surface is zero; (4) The membrane surface is impermeable and no-slip to fluid, indicating zero velocity. The corresponding equations are:

$$J_{2}\cdot n=0,\;J_{3}\cdot n=0,\;C_{1}=2mM,\;\Phi =0,U=0$$ (6)

At the microchannel walls, the boundary conditions are: (1) constant surface charge density; (2) no-slip condition for fluid velocity; (3) impermeability to ions and particles. The corresponding equations are:

$$\sigma_{-}=-1\text{mC/}{\text{m}}^{2},\;U=0,\;J_{i}\cdot n=0,i=1,2,3$$ (7)

At the inlet boundary, the boundary conditions are: (1) both the concentration of the electrolyte and particles are the same as those in the inlet reservoir. (2) the electric potential is *V*_*L*_; (3) the pressure is zero. The corresponding equations are:

$$\Phi =V_{L},\nabla U\cdot n=0,\;P=0,\;C_{i}=C_{i,0},\;i=1,2,3$$ (8)

At outlet boundary, the boundary conditions are: (1) free boundary conditions are applied for fluid flow; (2) the electric potential is set to *V*_*R*_. The corresponding equations are:

$$\Phi =V_{R},\;\nabla U\cdot n=0,\;P=0,\;\nabla C_{i}\cdot n=0,\;i=1,2,3$$ (9)

At the reservoirs walls, the boundary conditions are: (1) no-slip condition for fluid velocity; (2) zero charge. The corresponding equations are:

U=0,∇Φ⋅n=0. (10)

Governing equations (1) through (5) are solved using COMSOL^®^ (v5.2a), with the respective boundary conditions. Both time-dependent and stationary studies were conducted to investigate the transient and steady-state behaviors of the system.

To directly compare the experimental results in Fig. 2 and simulation, we plot the simulated concentrations of DNA and BSA at different times in the full-width channel in Fig. 4(a) and Fig. 4(b), respectively. Similar to the experiments, the concentration of DNA forms a typical profile of small biomolecules in the microchannel: a band with almost equal concentration over the width, growing from boundary of the depletion zone before membrane and expanding to the upstream channel. The system reaches the steady state in about 2×10^4^ s, with the maximum DNA concentration of 0.032 mM, corresponding to an enrichment factor of 3.2×10^4^. For BSA, protrusions into the depletion zone are formed. The system reaches the steady state at about *t*=250 s, with the maximum BSA concentration of 5.33×10^-3^ mM and an enrichment factor of 5330. It could be easily seen that, the trapping efficiency of BSA, in terms of the amount of particles focused, is significantly smaller than that of DNA. Although Fig. 2 and Fig. 4 show dramatically different time scales (simply because of the difference in 2D and 3D systems), key signatures of the profiles are faithfully reproduced in 2D.

**FIG. 4.**
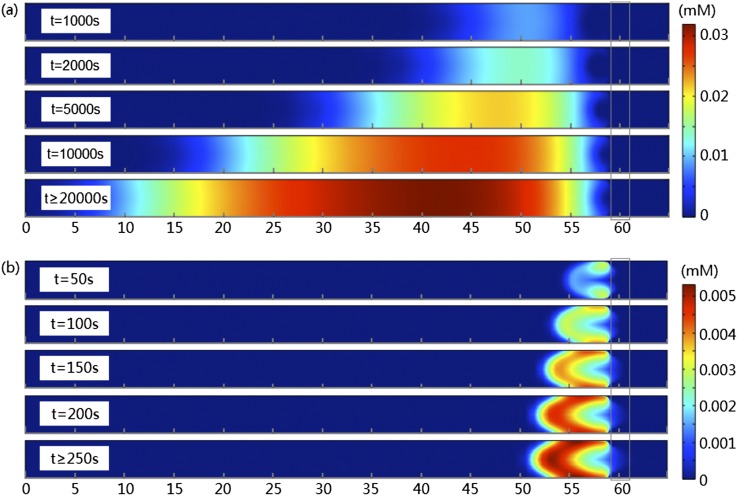
Concentrations of (a) DNA and (b) BSA at different times. The gray box at 59-61μm shows the location of the membrane.

To understand the significant differences in the trapping behaviors of BSA and DNA, we analyzed the interplay between the electric forces and the fluid drag forces experienced by these particles. Because the steady states define the ultimate behaviors of different systems, the following analyses will be focused on the steady states only. To highlight the effects of different particles in the behavior of their respective systems, we will also show the results of the system under the same conditions but with only the KCl solution, without any particles.

Figs. 5(a) though 5(c) show the electric fields in the microchannels filled with KCl, KCl+DNA and KCl+BSA, respectively. Electric fields in the upstream are generally in the *x*-direction, while electric fields in the downstream channel is mainly in the *y*-direction. From the differences between the smooth electric field in Fig. 5(a) and the distorted fields in Fig. 5(b) and Fig. 5(c), we know that focused DNA or BSA molecules deform the electric field significantly, especially near the membrane region. The strongest electric fields occur near the membrane surface because of the ICP. The strong electric fields and high (positive) extended space charge densities induced by ICP^22^ generate strong electric forces in the fluids in that region. While vertical component of the electric forces increases the pressure near the channel and membranes surfaces, horizontal component of electric force speeds up the rightward fluid flow there. Because the maximum horizontal force occurs before the intersection of the membrane and the channel wall in the upstream microchannel (*x*=59μm), the fluid is most accelerated there. This increases the pressure on the right side and deceases it on the left side, which induces the backflow of fluid in the center regions of the channels. One pair of circulating vortices are therefore generated (Fig. 6 shows only the counter-clockwise one in the lower half channel) in each case.^18^ One important characteristic of the flow field of incompressible fluid is that the streamlines cannot intersect. As a result, the flow fields in the microchannel can be decomposed into a laminar flow from the inlet to the outlet and a rotating vortex. If diffusion is not considered, the fluid that enter the microchannel from the inlet would pass through the membrane surface region (at high velocity) without entering the vortex. Meanwhile, the fluid inside the vortex would be trapped inside the vortex permanently. Charged particles may move across these streamlines driven by the electric field and thermal diffusion.

**FIG. 5.**
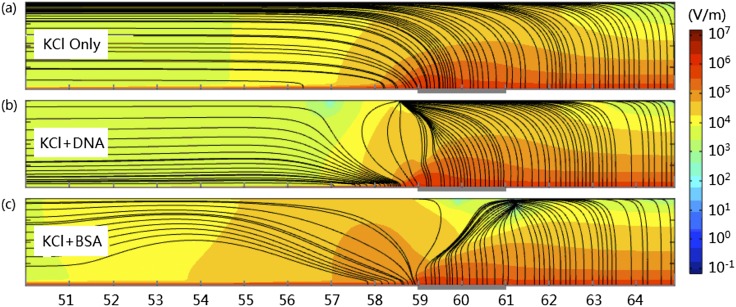
Electric field in the lower half of the microchannel of solutions of (a) KCl only, (b) KCl+DNA, and (c) KCl+BSA. Colors in background depict the magnitude of the electric field in logarithm scale. The black streamlines show the direction of the electric field.

**FIG. 6.**
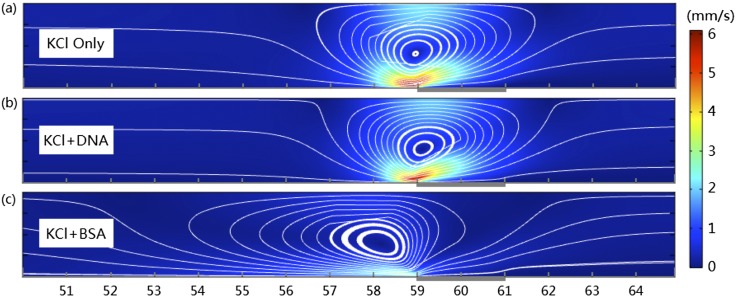
Streamlines of vortical flows near the membrane regions in the lower half of the microchannels filled with (a) KCl only, (b) KCl+DNA, and (c) KCl+BSA. Colors in the background show the magnitude of fluid velocity.

The electric fields **E** in Fig. 5 and the flow fields **U** in Fig. 6 determine the electric forces and drag forces on charged species inside the microchannel. According to the Stokes-Einstein relation,^28^ for charged species *i* with diffusion coefficient *D*_*i*_, the electric force and the fluid drag force applied to one mole of stationary particles are **F**_**E**_ = *Z*_*i*_*F***E** and **F**_**D**_ = *RT***U**/*D*_*i*_, respectively. Please note that this drag force is defined with respect to a fixed particle, which will result in a velocity **U** of the particle with reference to the fixed space (the same as that of the fluid). The total forces **F** = **F**_**E**_ + **F**_**D**_ applied to Cl^-^ (for KCl only), DNA and BSA are shown in Fig. 7. It could be found that the force applied to Cl^-^ is dominated by the electric field (c.f. streamlines of **E** in Fig. 5(a)), with the minor contribution from the fluid flow. In contrast, the force field experienced by BSA is dominated by the fluid flow (c.f. streamlines of **U** in Fig. 6(c)). The force field of DNA is affected by both. In all cases, the strong electric forces above the membrane push all the negatively charged species up into the center of the channel. For Cl^-^ in Fig. 7(a), it will turn to the upstream, leaving a depleted zone near the membrane region. In contrast, most DNA molecules follows the recirculated fluid flow, turns to left near the surface of channel wall and finally becomes focused in the upstream channel, while a small portion of DNA molecules circulate around the center of fluid vortex near the membrane. On the other hand, BSA molecules join the streamlines of the fluid vortex and are immediately transported back to the region near the channel surface before the membrane. Most BSA molecules are circulated in that region.

**FIG. 7.**
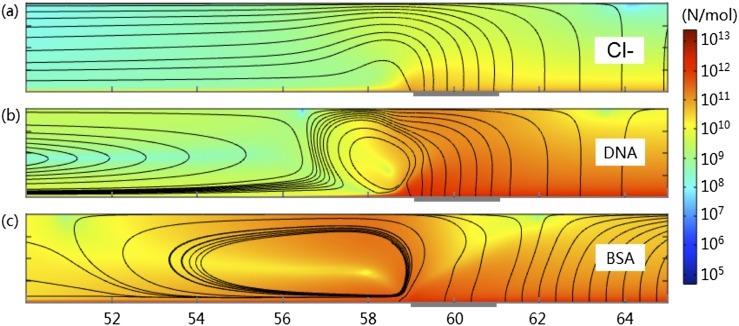
Total forces experienced by (a) Cl^-^ in KCl solution; (b) DNA in KCl+DNA; and (c) BSA in KCl+BSA. The background colors show the magnitudes of total forces in logarithm scale. The streamlines give the directions. Only the lower half of the channel is plotted.

Besides the deterministic force fields as shown in Fig. 7, random thermal motions of ions and particles contribute to their transport behaviors in the microchannel as well. Putting together the electrophoretic flux induced by electric field, convective flux by fluid flow and diffusive flux by thermal diffusion, the total flux densities of different charged species are obtained as shown in Fig. 8. Here the background color shows the concentration of Cl^-^ (for KCl only) or particles with respect to their respective ranges (c.f. Fig. 4). The lengths of the arrows are scaled by the tenth root of the magnitudes of their original fluxes, in order to make some of small fluxes visible. From the lengths of the arrows we found that the fluxes of Cl^-^ are significantly smaller than those of DNA and BSA (note that an arrow of double length corresponds to the flux of 2^10^ times). The flux of DNA molecules to the downstream channel is negligible, meaning that most DNA molecules are trapped. In contrast, there are non-negligible fluxes of BSA that leaked through the membrane region to the downstream microchannel, carried by the fluids passing through the membrane surface (c.f. Fig. 6(c)). This is the reason why the concentration factor of BSA is low: the portion of the particles that are sent to the inner circle of the vortex by the electric force near the membrane region is small. To quantify this effect, we calculated the integration of rightward fluxes (*J*_*3,x*_*>*0) of BSA and DNA over the cross section at different channel locations. We found 1/10^4^ of BSA molecules that arrive at the vortex region leaked through the membrane surface and migrated to downstream channel, while this ratio for DNA is in the order of 10^-17^. Another important feature provided by Fig. 8 is that larger particles are undergoing faster circulation. From Fig. 8(b) and Fig. 8(c), we found that the magnitude of fluxes of DNA and BSA is similar in their respective focused regions. Because maximum concentration of DNA (∼0.03 mM) is about 6 times of BSA (∼0.005 mM), the velocities of BSA have to be 6 times larger than DNA. That is why the band of BSA is very unstable.

**FIG. 8.**
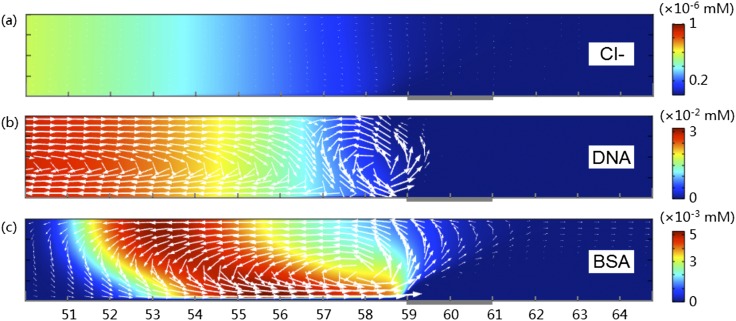
Fluxes of (a) Cl^-^ in KCl solution, (b) DNA in KCl+DNA, and (c) BSA in KCl+BSA. The background colors show the concentration of respective species in their own scale. The lengths of the arrows are scaled by the tenth root of the magnitudes of their original fluxes densities. Only the lower half of the channel is plotted.

In summary, this letter analyzes a detailed picture of force fields in the micro-nano-fluidic preconcentration system through 2D numerical simulation. Force fields of BSA (large molecule), DNA (small molecule), and the Cl^-^ ions (without particles) are analyzed, in terms of the interplay between the electric fields and the fluid flow characteristics. Features that are not explainable in 1D, namely the *protrusions* in focused band and the mechanism behind the low enrichment factor and instability of focused band of large particles, are clarified. Although 2D results are not expected to match the experimental data directly, qualitative mechanisms and physical insights obtained here may help researchers better understand, design and optimize ICP-based preconcentrators for mixed species,^29,30^ as well as devices for desalination,^31–33^ separation,^25,34^ reaction,^35–37^ micro-nano-fluidic mixing,^38,39^ and other related applications.^12,40^
